# Diet Induced Mild Hypercholesterolemia in Pigs: Local and Systemic Inflammation, Effects on Vascular Injury – Rescue by High-Dose Statin Treatment

**DOI:** 10.1371/journal.pone.0080588

**Published:** 2013-11-15

**Authors:** Marco Busnelli, Stefano Manzini, Alberto Froio, Alessia Vargiolu, Maria Grazia Cerrito, Ryszard T. Smolenski, Massimo Giunti, Alessandro Cinti, Augusta Zannoni, Biagio Eugenio Leone, Monica Forni, Maria Laura Bacci, Giorgio Maria Biasi, Roberto Giovannoni, Marialuisa Lavitrano

**Affiliations:** 1 Department of Surgery and Interdisciplinary Medicine, University of Milano-Bicocca, Milano, Italy; 2 Department of Biochemistry, Medical University of Gdansk, Gdańsk, Poland; 3 Department of Veterinary Medical Science, University of Bologna, Ozzano dell’Emilia, Bologna, Italy; University of Milan, Italy

## Abstract

**Objective:**

The aim of the present study was to comprehensively evaluate systemic and local inflammation as well as progression of vascular inflammation in normal and mechanically injured vessels in a large animal model of mild hypercholesterolemia. Our aim was also to test the effect of high-dose statin treatment on these processes.

**Methods:**

Pigs were kept for 120 days on a standard diet (SD, n=7), high-cholesterol diet (HCD, n=7) or high-cholesterol diet with Atorvastatin starting after 50 days (STATIN, n=7). Left carotid artery balloon injury was conducted in all groups after 60 days of diet treatment. Biochemical analysis together with evaluation of blood and tissue markers of vascular injury and inflammation were performed in all groups at the end of experiment.

**Results:**

HCD compared to SD induced systemic inflammation demonstrated by increased number of circulating monocytes and lymphocytes. HCD compared to SD induced also local inflammation demonstrated by adipocyte hypertrophy and infiltration of T-lymphocytes in abdominal white adipose tissue, activation of hepatic stellate cells with infiltration of T- and B-lymphocytes and macrophages in the liver and increased macrophage content in lung parenchyma. These changes were accompanied by increased Intima/Media thickness, stenosis, matrix deposition and activated T-cell infiltrates in injured but not in uninjured contralateral carotid artery as we previously reported. The treatment with high-dose statin attenuated all aspects of systemic and local inflammation as well as pathological changes in injured carotid artery.

**Conclusions:**

Diet related mild hypercholesterolemia induce systemic and local inflammation in the liver, lung and adipose tissue that coincide with enhanced inflammation of injured vessel but is without deleterious effect on uninjured vessels. High dose statin attenuated systemic and local inflammation and protected injured vessels. However, finding exact role of reduced systemic and remote inflammation in vascular protection requires further studies.

## Introduction

The long-term consequences of high-fat and/or high-cholesterol diet consumption are associated with an increased risk of cardiovascular disease, fatty liver disease, obesity and type 2 diabetes [1].The fatty streak and atheroma are the most prominent alterations associated with high cholesterol diet. Most severe consequence of high cholesterol diet is, however, development of vascular changes. Our group and others demonstrated that deleterious effect of hypercholesterolemia is particularly evident in vessels injured by other factors such as mechanical stress during surgery [2,3]. Neointimal hyperplasia after vascular injury mainly consists of a proliferative response of smooth muscle cells, deposition of extracellular matrix, (ECM), systemic and local inflammation. Neointimal hyperplasia plays a decisive role in restenosis, a process actively sustained by the proliferation and migration of vascular smooth muscle cells (VSMCs) in response to various inflammatory stimuli. The change of phenotype of VSMCs results in capability to migrate and synthesize ECM. 

Metabolic cells that are exposed to excess of nutrients and energy respond triggering a chronic inflammation not only in vasculature but also in other organs. The architecture of liver, white adipose tissue (WAT) and lung is characterized by a close interaction between metabolic and immune cells. In obese individuals, cells of the metabolic tissues (such as adipose tissue and liver) can eventually initiate the pro-inflammatory signaling cascade causing activation of leukocytes, thus leading to tissue-specific inflammation [4]. 

Recent evidence in mice suggested that dietary cholesterol exacerbates inflammatory changes due to an increased recruitment of leukocytes in WAT, particularly macrophages and T-lymphocytes [5–7]. Over time, WAT inflammation leads to the induction of cytokines such as tumor necrosis factor alpha (TNF-α), interleukin-1 beta (IL-1β) and interleukin 6 (IL-6) with further recruitment of immune cells resulting in a stronger proinflammatory response [8]. High cholesterol loads result in a proinflammatory phenotype with activation of hepatic inflammatory genes and recruitment of several leukocyte subsets [5,9,10]. Cholesterol crystals have been found in early diet-induced atherosclerotic lesions and it seems that these crystals could play a key role in triggering inflammatory response [11,12]. Interestingly, liver inflammation can develop independently of steatosis upon high-cholesterol feeding. Hence, it has been proven that the removal of cholesterol from the diet prevents hepatic inflammation without affecting steatosis [13]. The lung parenchyma contains alveolar macrophages derived from blood monocytes that patrol the airways to engulf foreign particles. In mice, hypercholesterolemia can increase the number of infiltrating pro-inflammatory macrophages associated with lung remodeling; in addition, hypercholesterolemia is considered a potential risk factor for asthma [14,15].

Heme oxygenase 1 (HO-1) is a stress-induced protein that is expressed in response to a variety of stimuli. HO-1 expression is implicated in protection against atherosclerosis. Previous studies have shown that the increase in HO-1 expression and activity by statins may have an anti-atherosclerotic effect in humans and in animal models of atherosclerosis [16,17].. HO-1 seems to be involved also in the regulation of nitric oxide synthase 2, inducible (iNOS) expression and nitric oxide (NO) production. Recent studies have shown that iNOS may be expressed in the human atherosclerotic plaque. Indeed, one of the hallmarks of a dysfunctional endothelium is diminished levels of bioavailable NO. Oral administration of L-Arginine, the precursor of NO, reduces neointimal hyperplasia in balloon-injured rat carotid arteries [18,19].

Statins, by inhibiting 3-hydroxy-3-methylglutaryl-coenzyme A (HMGCoA) reductase, decrease endogenous cholesterol synthesis. Most data indicate that statins exert pleiotropic effects in addition to the lowering of serum cholesterol [20]. These include a broad range of anti-inflammatory mechanisms that inhibit leukocytes motility and adhesion to the vascular wall reducing the amount of tissue-infiltrating leukocytes [21], associated to a significantly decreased white blood cells (WBCs) count [22,23].

Mouse models have been widely used to evaluate the effects of high-fat feeding, however many of the evidences obtained are biased by differences in anatomy, lipid metabolism and lipoprotein profile. Unlike humans, even an excess of blood cholesterol in mice is carried by high-density lipoprotein (HDL) and not by low-density lipoprotein (LDL), this could hamper the translation of experimental results obtained in mice to humans [24]. On the contrary, we and others have previously reported that the pig model could help to provide insights into metabolic and cardiovascular diseases because pigs share with humans several aspects of lipoprotein metabolism and the lipoprotein profile. In particular, when pigs were fed with high levels of saturated fat and cholesterol, plasma total cholesterol increased and complex atherosclerotic lesions, with features similar to those seen in humans, appeared [2,25,26]. Clinical studies concerning dietary cholesterol have focused on its effect on plasma lipids and lipoproteins without investigating the effect of hypercholesterolemia on tissue-specific inflammation. 

In the present study we evaluated for the first time in a porcine model of mild-hypercholesterolemia whether high-cholesterol diet, besides vascular changes, is sufficient to induce a systemic and tissue-specific inflammation. We addressed the effects of high-dose atorvastatin treatment on entire spectrum of vascular, systemic and tissue specific inflammation to identify key factors involved in pathology. 

## Methods

### Animals

Large White female pigs (n=21) were purchased from a commercial breeder. Experiments involving animals were carried out according to a protocol approved by the Animal Care Committee of the Italian Minister of Public Health. The investigation conformed to the Guide for the Care and Use of Laboratory Animals published by the US National Institutes of Health (NIH Publication No. 85-23, revised 1996).

### Experimental Protocol

Pigs were allocated into three groups: SD (n=7), pigs fed a standard porcine diet (3.6% lipid content); HCD (n=7), pigs fed a high-cholesterol diet (27% lipid content: 5% cholesterol and 22% beef tallow); STATIN (n=7), high-cholesterol fed pigs (the same diet as for HCD group) treated orally with high-dose atorvastatin (Torvast; Pfizer) starting after 50 days of high-cholesterol diet (80 mg/day/55Kg pig) until the end of the experimental procedure (average weight of pigs, about 70Kg). HCD-fed pigs received nearly 1100 Kcal extra/day compared to SD-fed pigs. The diet had an overall duration of 120 days.

After 60 days, the vascular injury in porcine carotid was induced as previously described [2].

### Laboratory tests

Blood samples were collected from pigs at the beginning of diet administration and at sacrifice. The plasma obtained was kept at -80°C until the measurements. Aspartate aminotransferase (AST), Alanine aminotransferase (ALT), total, LDL and HDL cholesterol and glucose were determined with colorimetric/enzymatic assays (Olympus system reagent, Olympus, Milan, Italy). All biochemical assays were carried out on an automated analyzer (Olympus AU 400). To determine counts of red blood cells, hemoglobin and hematocrit levels, WBCs, monocytes, lymphocytes, neutrophils, basophils, eosinophils, and platelets, complete blood counts were performed on EDTA anticoagulated blood samples using a Cell-Dyn 3500 system (Abbott Diagnostics). Hemograms were performed using a Cell-Dyn 3500 system (Abbott Diagnostics). Blood smears were May-Grunwald-Giemsa stained and visually evaluated for white blood cell differential counts. Analyses of inflammatory cytokines were performed using the SearchLight Chemiluminescent Array Kit specific for IL-1β, IL-6, interferon-gamma (IFNγ) and TNFα (Pierce Biotechnology). Assays were performed according to the manufacturer’s instructions. 

### Histological analysis and immunohistochemistry

Balloon-injured left carotid arteries and contralateral carotid arteries (as negative control) as well as liver, lung and subcutaneous abdominal WAT were harvested at sacrifice, fixed in paraformaldehyde 4% and embedded in paraffin blocks. Serial sections (3 µm thickness) were cut and either stained with hematoxylin&eosin or Masson’s Trichrome staining (Bio-Optica).

Immunohistochemical staining was performed on paraffin-embedded tissue sections of carotid arteries, liver, lung and subcutaneous abdominal WAT from each pig. To test antibody specificity, for each primary antibody used, control sections were incubated a) without the primary antibody or b) with the antibody, which had been previously incubated in presence of the peptide antigen (when commercially available). Antigen retrieval was achieved by boiling in citrate buffer (pH 6.0). Tissue sections were incubated with primary antibodies for staining the following antigens: mouse anti-human CD45RO (M0742) DAKO, dilution 1:200; mouse anti-human MAC387 (sc-66204), Santa Cruz, dilution 1:200; mouse anti-human CD20 (18-0088), Zymed, dilution 1:300; mouse anti-human CD15 (MY1), Histo-Line Laboratories, dilution 1:100; mouse anti-human CD138 (MI15), DAKO, dilution 1:100; mouse anti-human smooth muscle actin (MS-113-P), Neomarkers, dilution 1:3000; mouse anti-human type I collagen (sc-59772), Santa Cruz, dilution 1:400; rabbit anti-porcine TGF-β1 (sc-146), Santa Cruz, dilution 1:250; rabbit anti-human TGFβRII (sc-400), Santa Cruz, dilution 1:50; rabbit anti-human HO-1 (spa-895), Stressgen, dilution 1:1000; rabbit anti-human iNOS (sc-651), Santa Cruz, dilution 1:200. VSMCs in carotid arteries have been detected using an antibody specific for the alpha smooth muscle actin (ab5694, Abcam, dilution 1:200). Proliferation index was evaluated by immunohistochemistry with a mouse monoclonal anti-Ki67 antibody (MM1, Vector Laboratories, dilution 1:100). Apoptosis index was evaluated by immunohistochemistry with a rabbit polyclonal antibody specific for the cleaved form of caspase-3 (Asp175, Cell Signaling Technology, dilution 1:50). A biotinylated secondary antibody was used for streptavidine-biotin-complex peroxidase staining (Vectastain Abc Kit, Vector Laboratories). DAB was used as chromogen (Sigma-Aldrich), and sections were counterstained with hematoxylin (Mayer’s Hematoxylin, Lillie’s Modification, Dakocytomation). At least three slides per tissue per pig were analyzed for each staining. The Aperio ScanScope GL Slide Scanner (Aperio Technologies, Vista, CA, USA) system was used to capture digital images with a 40x scanning magnification (20x with 2x magnification changer). The ScanScope console was equipped with a Nikon 20x/0.75 Plan Apochromat objective producing a 0.25 µm/pixel scanning resolution. The Aperio ImageScope software (version 8.2.5.1263) was used to acquire and process digital images. 

### Morphometric analysis

The carotid arteries were harvested 60 days after the balloon-injury procedure. The carotid arteries were fixed in paraformaldeyde 4% and embedded in paraffin blocks. Serial sections (3 um thickness) were cut and stained with hematoxylin and eosin. Two independent operators (MB and AF) blindly evaluated all the sections obtained for each carotid artery by microscopic analysis. The ten sections showing the worst damage were selected for a full morphometric analysis, including: Intima-Media Area ratio, Intima-Media Thickness ratio, Intima-Media Thickness, degree of stenosis, intimal thickness, medial thickness, internal elastic lamina perimeter, external elastic lamina perimeter, internal elastic lamina area, intimal area, external elastic lamina area, medial area. The computer assisted analysis has been performed using specific software (Aperio, Imagescope). The degree of stenosis has been evaluated using the following formula: (Area within the internal elastic lamina – Lumen Area)/(Area within the internal elastic lamina)*100. The results reported for each animal for Intima/Media (I/M) Area ratio, I/M Thickness ratio, I/M Thickness and degree of stenosis represent the average value obtained by considering the three sections showing the worst damage. 

### Statistical analysis

Data are presented as the mean ±S.E.M. Datasets from each experiment were tested for normal distribution using SigmaPlot software. For studies involving two groups, comparisons were made using two-tailed t-tests. For multiple group comparisons were made using one-way ANOVA followed by Tuckey post-hoc test. Correlations were determined using Spearman’s rank order analysis. Differences were considered significant when *p*<0.05. The statistical analyses were done using SPSS 17.0.

## Results

### High-dose atorvastatin markedly reduces total serum cholesterol and LDL-cholesterol

We already reported that after 120 days the high-cholesterol diet significantly increased serum total cholesterol, LDL-cholesterol [2] (Table 1). The high-cholesterol diet treatment increased also triglycerides as compared to SD (Table 1). The HCD pigs showed a tendency towards an increased glycemia compared with the SD-fed pigs (Table 1). Treatment with high-dose atorvastatin significantly reduced serum total cholesterol and LDL-cholesterol compared to the HCD (Table 1), with a significant increase in HDL-cholesterol compared to the SD. Atorvastatin treatment neither reduced triglyceridemia nor glycemia compared to the HCD (Table 1).

**Table 1:** Serum parameters in SD-fed pigs, HCD-fed pigs and HCD-fed pigs treated with atorvastatin.

	**SD (n=7)**	**HCD (n=7)**	**HCD + Atorvastatin (n=7)**
**Total Cholesterol (mg/dL)**	65.4 ± 2.7^*^	89.9 ± 5.1	73.2 ± 3.1^*^
**LDL Cholesterol (mg/dL)**	37.9 ± 2.1^*^	56.5 ± 5.9	44.1 ± 4.3^*^
**HDL Cholesterol (mg/dL)**	24.2 ± 1.6	28.1 ± 2.6	32.1 ± 1^†^
**Triglycerides (mg/dL)**	20.1 ± 6.7^*^	48.6 ± 25.5	38.7 ± 12.3
**Glucose (mg/dL)**	89.9 ± 19.2	123.4 ± 53.8	126.7 ± 39.5^†^
**ALT (U/I)**	30.7 ± 9.3	23.1 ± 9.7	24.8 ± 2.3
**AST (U/I)**	22.0 ± 5.6	18.7 ± 6.8	20.8 ± 2.6
**TNF- (pg/mL)**	< 3	4.98 ± 1.1^*^	< 3
**IL-1**β (**pg/mL**)	< 3	6.15 ± 1.2^*^	< 3
**IL-6 (pg/mL)**	< 3	< 3	< 3
**IFNγ (pg/mL)**	< 3	< 3	< 3

SD = Standard Diet; HCD = High Cholesterol Diet. Comparisons were made using one-way ANOVA followed by Tuckey post hoc test. * p < 0.05 versus HCD; † p < 0.05 versus SD.

### Hypercholesterolemia increases the amount of monocytes and lymphocytes

Blood analysis performed after 120 days showed a significantly increased number of total WBCs in HCD-fed pigs compared to SD-fed pigs (Table 2). Blood smear counts demonstrated that this increase depended on monocytes and lymphocytes with a non-significant increase in the number of circulating neutrophils. Compared to the findings in HCD pigs, treatment with atorvastatin significantly reduced the amount of circulating WBCs, monocytes and lymphocytes to levels resembling those found in SD-fed pigs (Table 2).

**Table 2:** Haematological parameters in SD-fed pigs, HCD-fed pigs and HCD-fed pigs treated with atorvastatin.

	**SD (n=7)**	**HCD (n=7)**	**HCD + Atorvastatin (n=7)**
**WBCs** (**x10^4^ cells/mm^3^**)	1.1 ± 0.1^*^	2.1 ± 0.4	1.3 ± 0.1^*^
**Monocytes** (**x10^3^ cells/mm^3^**)	0.2 ± 0.05^*^	0.6 ± 0.09	0.2 ± 0.09^*^
**Lymphocytes** (**x10^3^ cells/mm^3^**)	7.1 ± 0.7^*^	10.2 ± 1.2	8.3 ± 0.7
**Neutrophils** (**x10^3^ cells/mm^3^**)	4.1 ± 0.9	6.9 ± 1.7	5.5 ± 0.8
**Eosinophils** (**x10^3^ cells/mm^3^**)	0.1 ± 0.04	0.3 ± 0.07	0.3 ± 0.02
**Basophils** (**x10^3^ cells/mm^3^**)	0.05 ± 0.02	0.1 ± 0.08	0.05 ± 0.04
**Platelets** (**x10^4^ Plts/mm^3^**)	33.3 ± 3.2	40.1 ± 3.8	33.3 ± 3.5
**Mean Platelet Volume (fL)**	4.0 ± 0.1	4.3 ± 0.2	4.2 ± 0.4
**Red Blood Cells** (**x10^6^ cells/mm^3^**)	5.5 ± 0.3	5.8 ± 0.3	5.6 ± 0.5
**Hemoglobin (g/dL)**	9.7 ± 0.5	10.4 ± 0.5	10.5 ± 0.3
**Hematocrit (%)**	30.4 ± 1.5	31.2 ± 1.7	30.0 ± 1.0

SD = Standard Diet; HCD = High Cholesterol Diet. Comparisons were made using one-way ANOVA followed by Tuckey post hoc-test. *p < 0.05 versus HCD.

### Atorvastatin attenuates hypercholesterolemia-induced adipocyte hypertrophy and T-lymphocyte infiltration in subcutaneous abdominal adipose tissue

**Figure 1:**
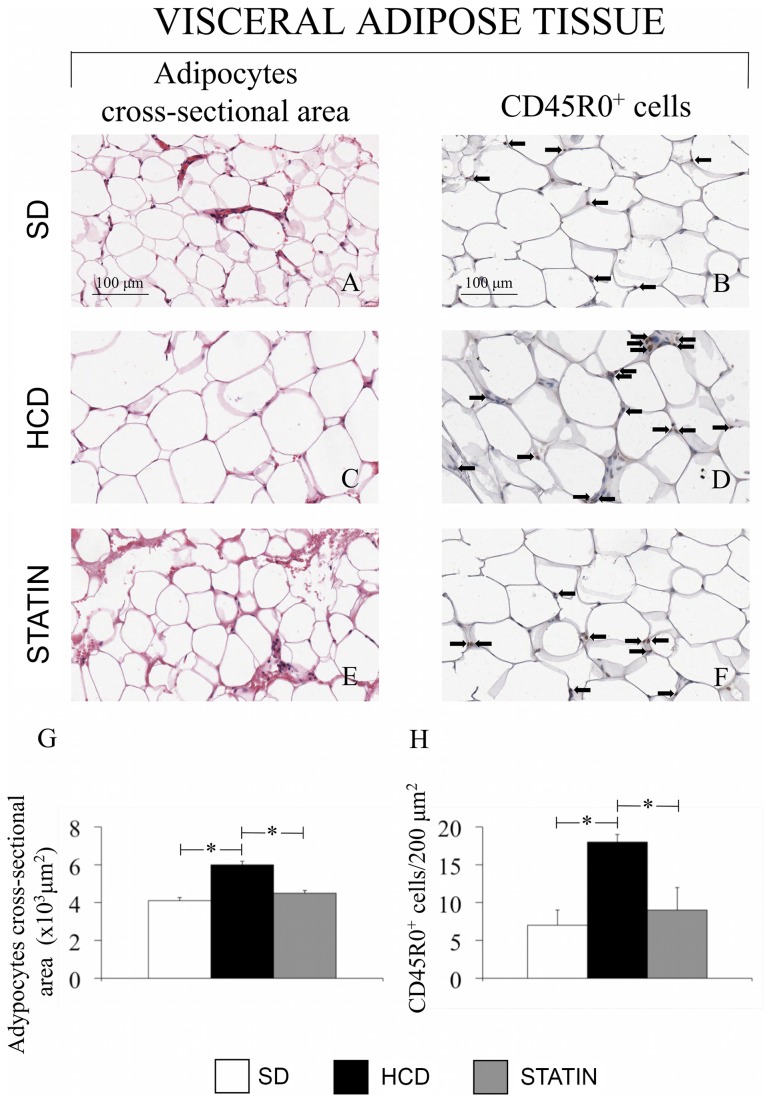
Adipocyte hypertrophy and T-lymphocyte infiltration in abdominal WAT of HCD-fed pigs. Hematoxylin and Eosin staining of abdominal subcutaneous WAT (A, C, E, G) and immunohistochemical staining specific for activated T-lymphocytes (CD45RO, B, D, F, H). Adipocyte area and T-lymphocytes are increased in HCD-fed pigs (C, D) compared with SD-fed (A, B) and atorvastatin-treated pigs (E, F). (Magnification 40x). *P<0.05.

Subcutaneous abdominal WAT from HCD-fed pigs showed adipocyte hypertrophy. The mean cross-sectional area of adipocytes from HCD-fed pigs was significantly increased compared to the adipocytes from SD-fed pigs. An immunohistochemical staining specific for activated T lymphocytes showed that high-cholesterol diet induced a significant increase in the amount of infiltrating T-lymphocytes into the abdominal WAT compared to SD. Treatment with atorvastatin effectively reduced both the adipocyte mean cross-sectional area and the amount of infiltrating T-lymphocytes compared to HCD (Figure 1). The immunohistochemical staining specific for MAC387 in WAT did not reveal a significant increase in the number of macrophages in HCD-fed and SD-fed pigs or after the treatment with atorvastatin (data not shown). In addition, the circulating levels of TNF-α and IFN-γ were significantly higher in HCD-fed compared to SD-fed pigs while no variations in circulating levels of IL-1β and IL-6 were observed (Table 1). Atorvastatin treatment significantly decreased the amount of circulating pro-inflammatory cytokines IFN-γ and TNF-α. (Table 1)

### Atorvastatin prevents the hypercholesterolemia-induced hepatic inflammation by reducing the number of infiltrating leukocytes

**Figure 2:**
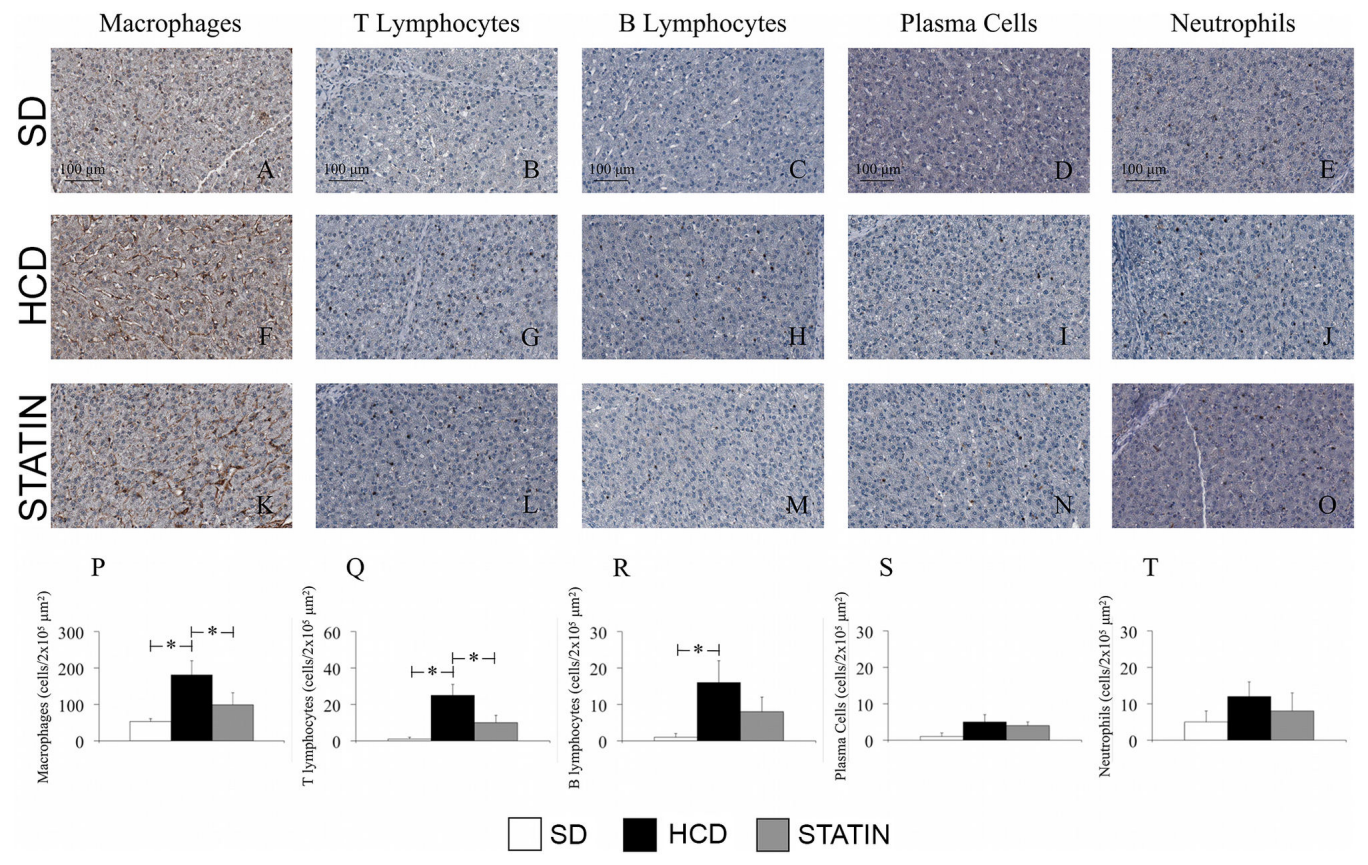
Increased numbers of macrophages, T- and B-lymphocytes in livers from hypercholesterolemic pigs. Immunohistochemical liver staining for macrophages (A, F, K, P), T-lymphocytes (B, G, L, Q), B-lymphocytes (C, H, M, R), plasma cells (D, I, N, S) and neutrophils (E, J, O, T). Infiltrating macrophages (A, F, P), T- (B, G, Q) and B-lymphocytes (C, H, R) but not plasma cells (D, I, S) and neutrophils (E, J, T) were significantly increased into the hepatic parenchyma of HCD-fed pigs compared with SD-fed pigs. The treatment of HCD-fed pigs with atorvastatin markedly reduced the amount of infiltrating macrophages (K, P) and T-lymphocytes (L, Q). (Magnification 40x). *P<0.05.

**Figure 3:**
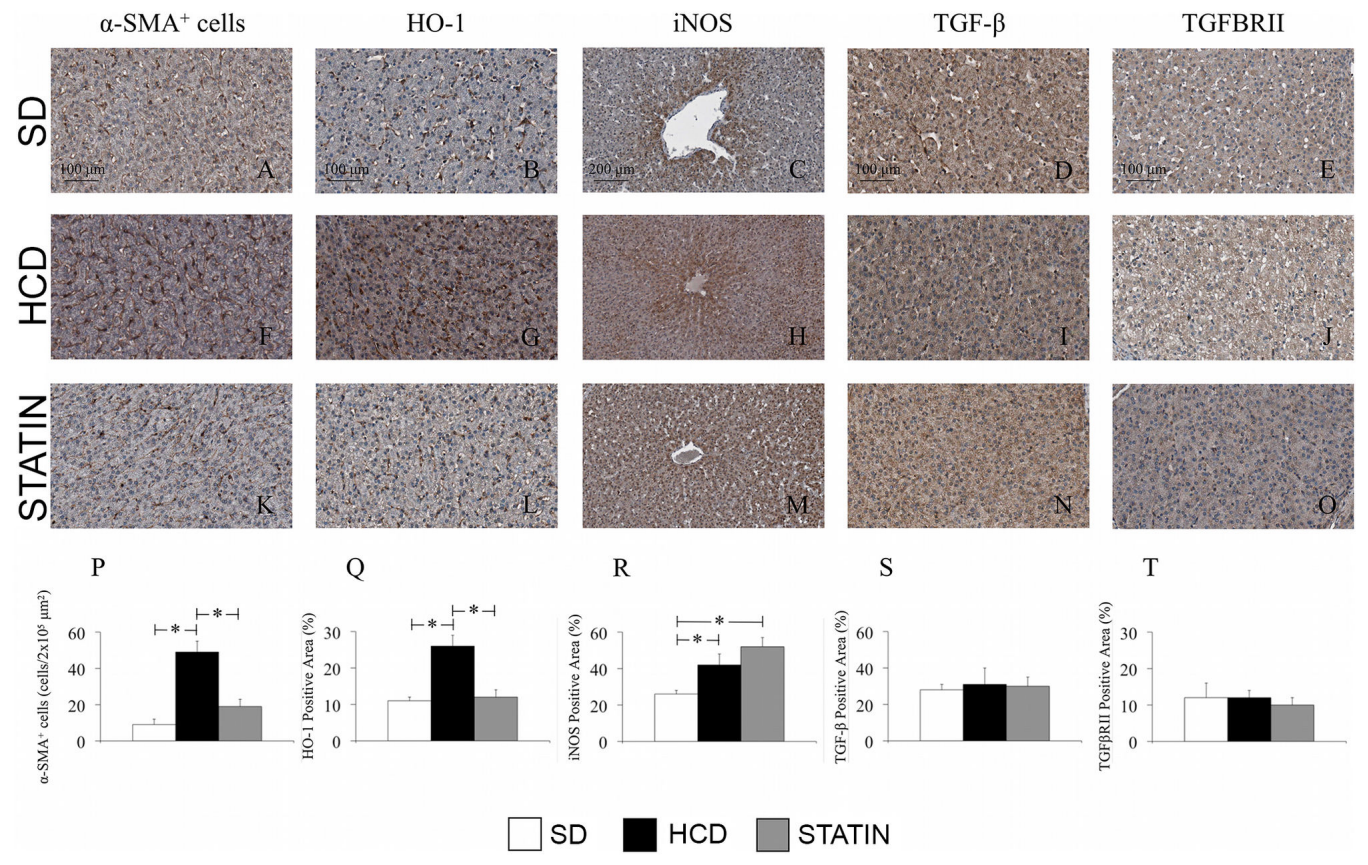
Atorvastatin reversed hypercholesterolemia-induced liver inflammation by lowering activated-HSCs and stimulating HO-1 and iNOS expression. Immunohistochemical liver staining for activated-HSCs (α-SMA, A, F, K, P), HO-1 (B, G, L, Q), iNOS (C, H, M, R), TGF-β1 (D, I, N, S) and TGFβRII (E, J, O, T). Hypercholesterolemia significantly increased the amount of activated HSCs (F, P), HO-1 (G, Q) and iNOS (H, R) expression without affecting TGF-β1 (I, S) and TGFβRII (J, T). Atorvastatin significantly reduced HSCs activation (K, P) and HO-1 expression (L, Q) but did not influence iNOS hepatic expression (M, R). (Magnification 40x).

**Figure 4:**
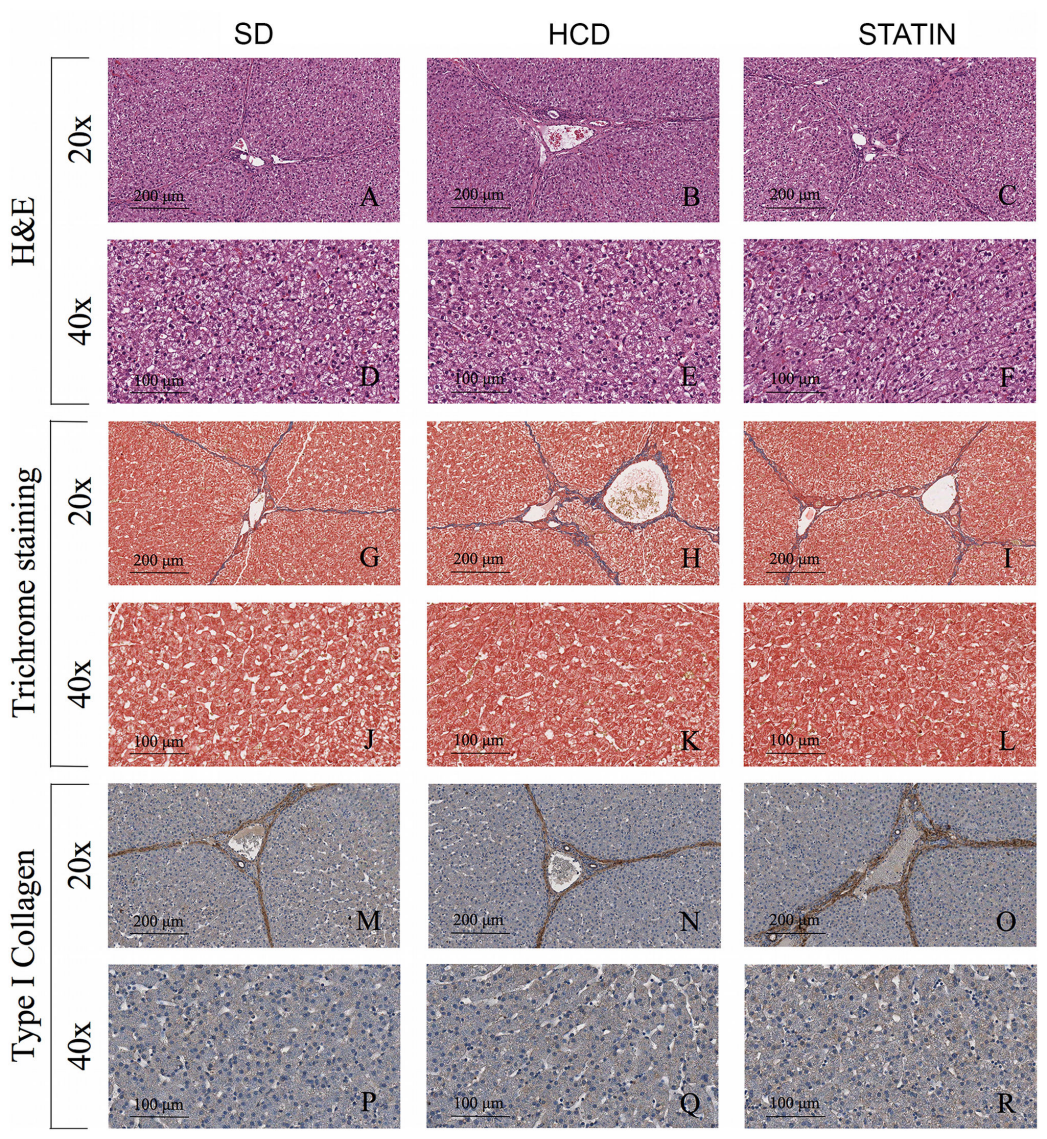
Hypercholesterolemia did not induce liver steatosis and fibrosis in HCD-fed pigs. Hematoxylin and Eosin staining (A-F), Masson’s trichrome staining (G-L) and immunohistochemical analysis for type I collagen (M-R) of livers from HCD-fed pigs showed neither signs of steatosis (B, E) nor fibrosis (H, K, N, Q) having a parenchymal structure and ECM deposition comparable with those found in SD-fed (A, D, G, J, M, P) and atorvastatin-treated pigs (C, F, I, L, O, R). *P<0.05.

Immunohistochemical liver analysis revealed increased leukocyte infiltration in the hepatic parenchyma of HCD-fed pigs compared with SD-fed pigs. The majority of leukocytes infiltrating the liver were macrophages (Figure 2). A significant increase in the amount of infiltrating T- and B-cells was also detected in livers from HCD-fed pigs compared with SD-fed pigs (Figure 2 B, G, Q and C, H, R). Non significant influxes of neutrophils and plasma cells were also seen in livers from hypercholesterolemic pigs compared to normocholesterolemic pigs (Figure 2 D, I, S and E, J, T). Atorvastatin treatment significantly decreased macrophage and T-lymphocyte infiltrates (Figure 2 F, K, P and G, L, Q), with a borderline reduction of B-lymphocytes (Figure 2 H, M, R). An immunohistochemical analysis on liver sections specific for alpha-actin (alpha-SMA) showed a significant increase in the number of alpha-SMA-positive cells in HCD-fed pigs compared with SD-fed pigs (Figure 3 A, F, P). There was a significant reduction of α-actin-expressing cells following atorvastatin treatment in comparison to the HCD subset (Figure 3 F, K, P). The histology of liver tissue from HCD-fed pigs did not display steatosis (Figure 4 A, F), notwithstanding the presence of an abundant leukocyte infiltrate with increased activation of hepatic stellate cells (HSCs). Moreover, there were no signs of fibrosis with no differences in ECM deposition (Figure 4 G, L), type I collagen content (Figure 4 M, R), TGF-β1 and TGFβRII expression between HCD-fed, SD-fed and atorvastatin-treated pigs (Figure 3 D, I, N, S and E, J, O, T). Consistent with the histological findings, ALT and AST levels were not increased in hypercholesterolemic pigs compared to SD-fed pigs (23.1 ± 9.7 vs. 30.7 ± 9.3 U/I, p=ns; 18.7 ± 6.8 vs. 22.0 ± 5.6 U/I, p=ns).

In HCD-fed pigs the expression of the stress-inducible genes HO-1 and iNOS was significantly increased compared to SD-fed pigs (Figure 3 B, G, Q and C, H, R). Atorvastatin had a different effect on HO-1 and iNOS, with a significantly decreased expression of HO-1 compared to the HCD subset (Figure 3 G, L, Q), but a further increase in iNOS expression compared to SD-fed pigs (Figure 3 H, M, R). 

### The high-cholesterol diet increases macrophage infiltration in lungs without lung remodeling

**Figure 5:**
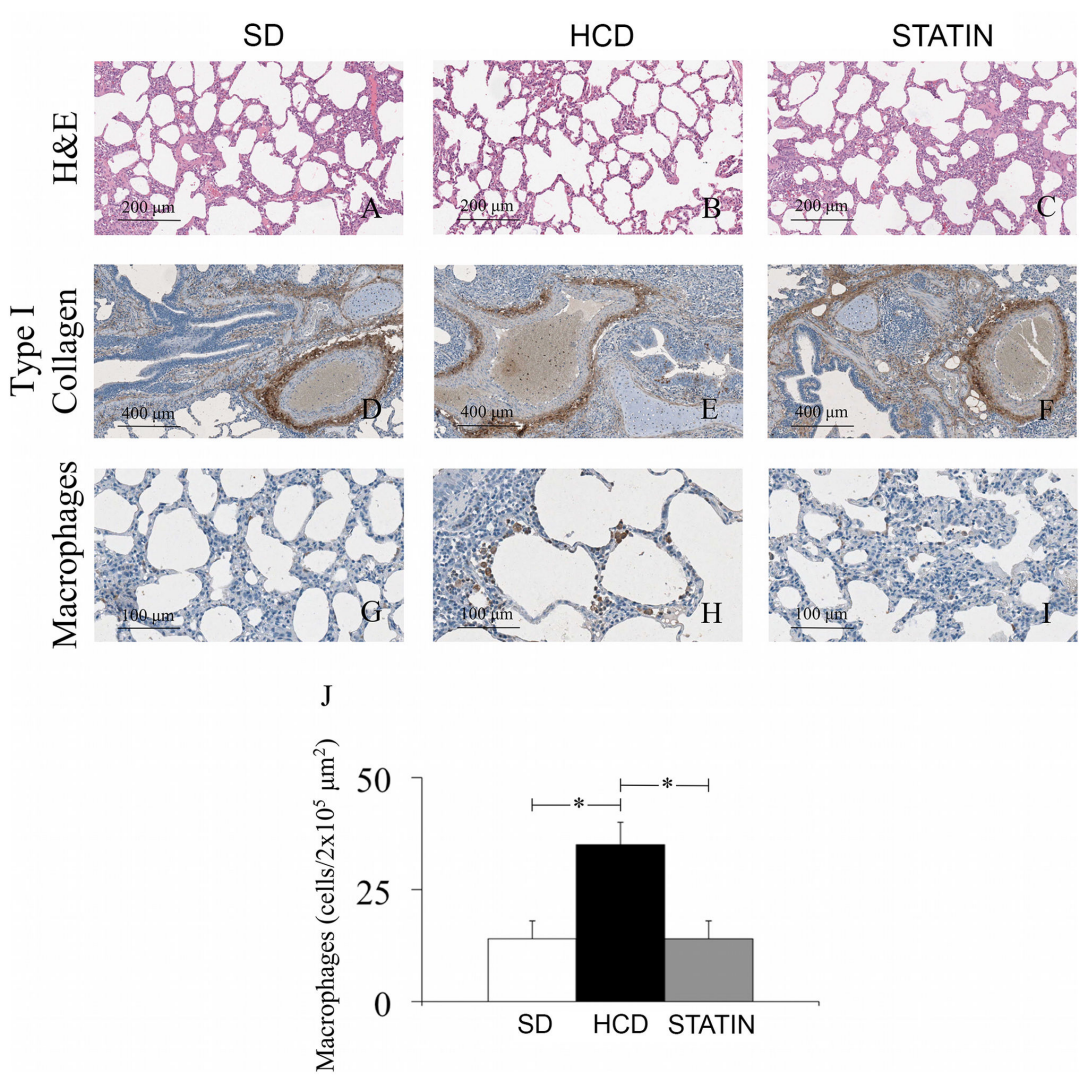
Increased macrophage infiltration but not tissue remodeling in lungs of hypercholesterolemic pigs. Hematoxylin and Eosin staining (A-C) and an immunohistochemical analysis specific for type I collagen (D-F) demonstrated that there were no signs of lung remodeling in HCD-fed pigs. The immunohistochemical staining specific for MAC387 showed a markedly increased number of macrophages in lungs from HCD-fed pigs (G, H, J). The treatment with atorvastatin dramatically reduced the number of infiltrating macrophages (I-J).

Lung sections from HCD-fed pigs showed a significantly increased recruitment of macrophages compared to the amount of macrophages detected in SD-fed pigs (Figure 5 G, H, J). The treatment with atorvastatin significantly reduced the number of infiltrating mononuclear cells (Figure 5 H, I, J). The amount of lymphocytes was also increased in lungs from HCD-fed pigs even though the increase was not statistically significant (data not shown). No changes in tissue integrity and ECM deposition were detected in lungs from HCD-fed pigs (Figure 5A-C), even if a chronic presence of inflammatory cells within the lungs is known to induce tissue remodeling. In accordance with these findings, immunohistochemical analysis on lung sections specific for type I collagen (Figure 5 D-F), TGF-β1 and TGFβRII expression revealed the same degree of ECM deposition independent of diet or treatment with atorvastatin.

### Protective effect of atorvastatin treatment in vascular injury

**Figure 6:**
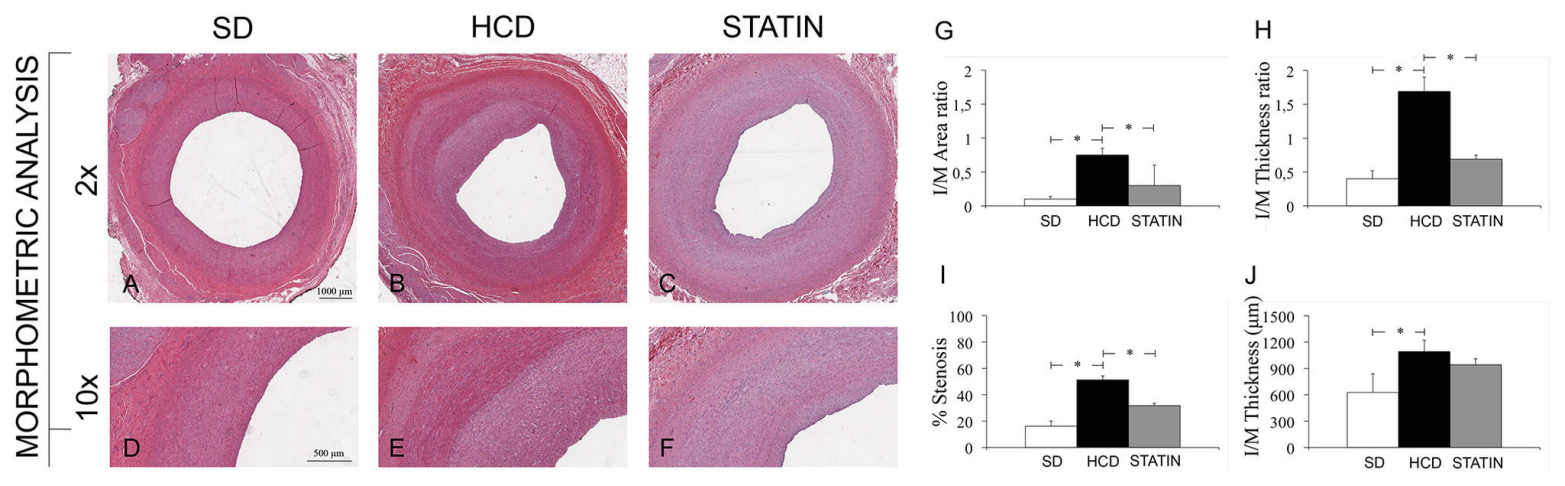
I/M Area and I/M Thickness ratio in injured and contralateral carotid arteries. Atorvastatin significantly reduced I/M Area and I/M Thickness ratio compared to HCD (B-C, E-F, G-H). The degree of stenosis was significantly greater in HCD compared to STATIN pigs (I). *P<0.05.

**Figure 7:**
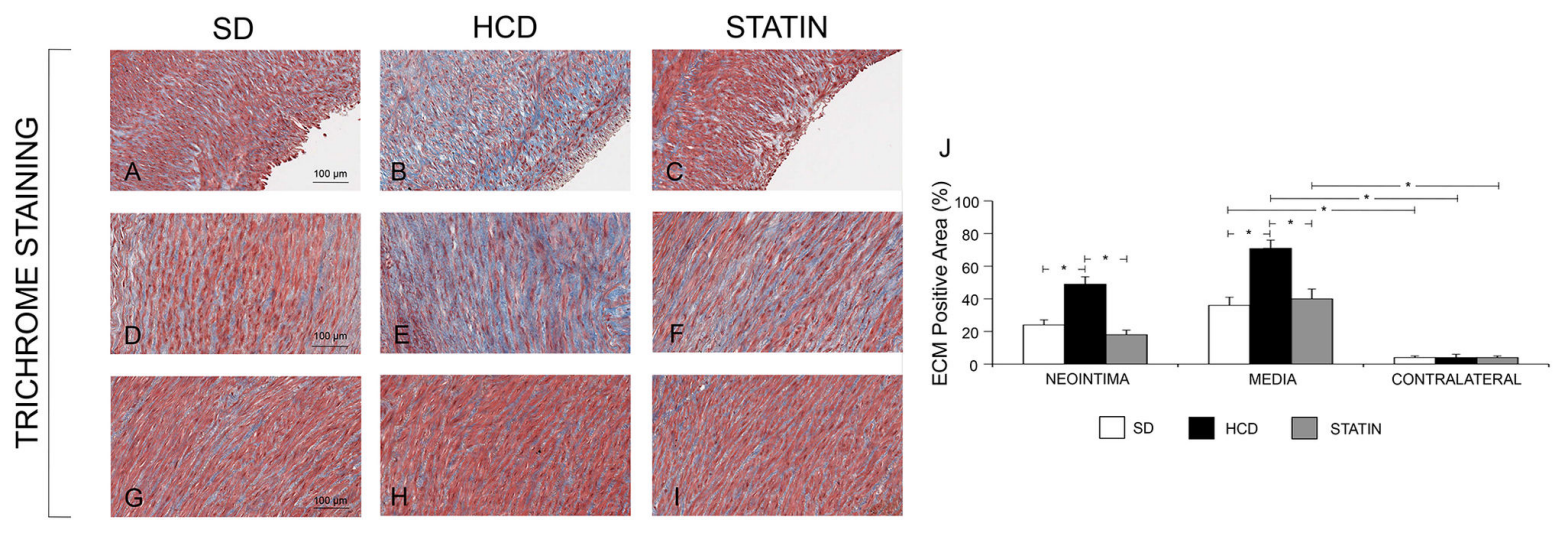
Hypercholesterolemia induced matrix deposition. The deposition of ECM was significantly increased in HCD compared to SD pigs, both in the neointima (A, B and J) and in the media (D, E and J). The deposition of ECM was significantly reduced in STATIN compared to HCD pigs, both in the neointima (C, J) and in the media (H, J). *P<0.05.

**Figure 8:**
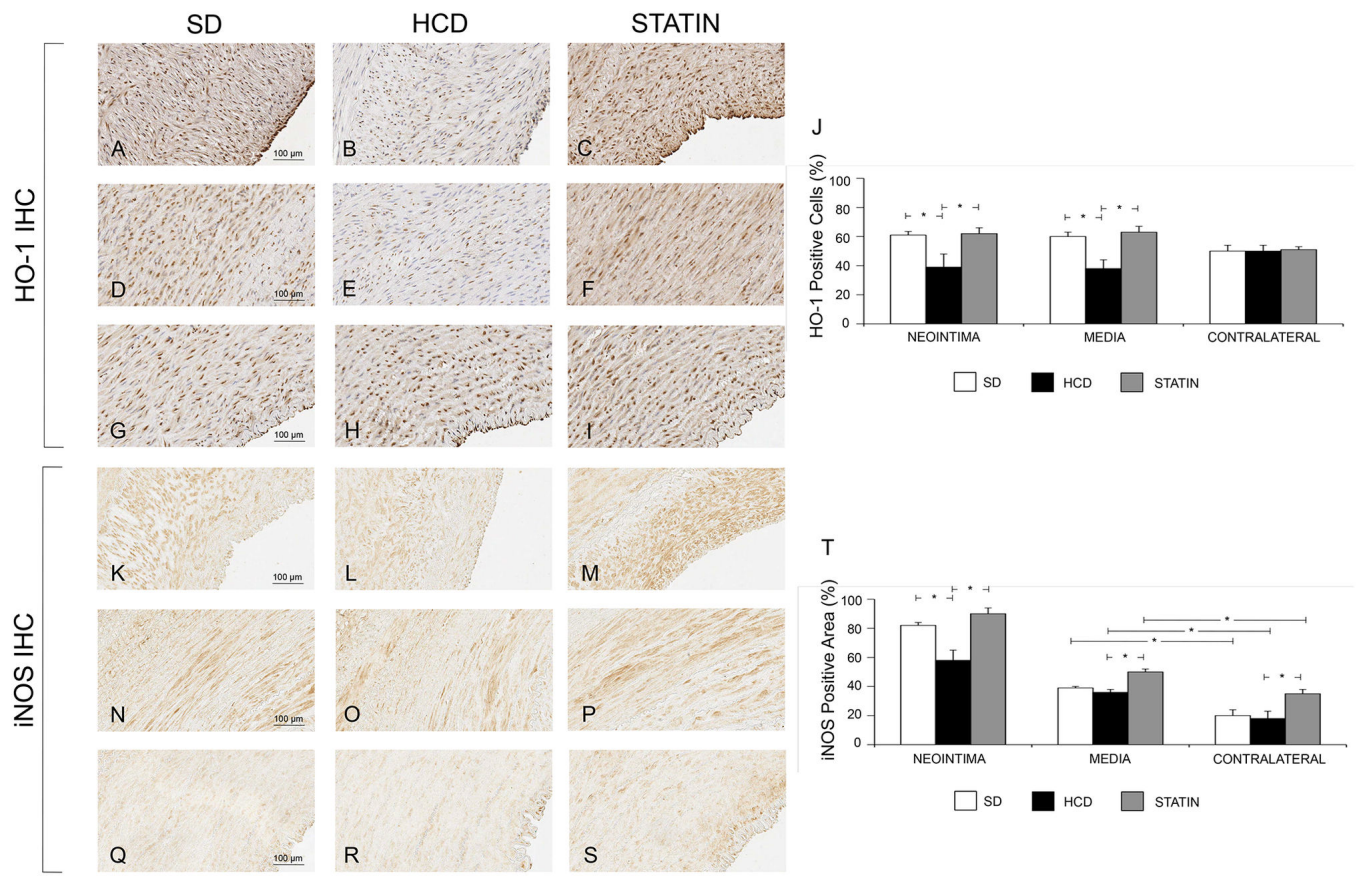
Atorvastatin restored the expression of the protective genes HO-1 and iNOS at the site of vascular injury, enhancing iNOS expression also in contralateral uninjured arteries. HO-1 expression was reduced in the neointima and injured media of CHOL compared to SD pigs (39±9% vs. 61±3, p=0.04, panels A, B and J, and 38±6 vs. 60±3, p=0.04,panels D, E and J). Atorvastatin treatment restored the expression of HO-1 to levels similar to SD group (62±4% vs. 39±9%, p=0.04, Figure A-C-J; and 63±4 vs. 38±6%, p=0.04, panels D, F and J). There were no differences in contralateral uninjured arteries (G, H, I and J). There was a significant reduction of iNOS expression following vascular injury in the neointima of HCD compared to SD pigs (58±7% vs. 82±2%, p=0.007, panels K, L and T). Atorvastatin treatment significantly restored the expression of iNOS in neointima compared to HCD (90±4% vs. 58±7%, p=0.005, panels K, M and T) and induced a significant expression in the media of injured and contralateral arteries in comparison to HCD and SD (50±2% vs. 39±1%, p=0.05 panels N, O, P and T; 35±3% vs. 18±5%, p=0.05, panels Q, R, S and T).

**Figure 9:**
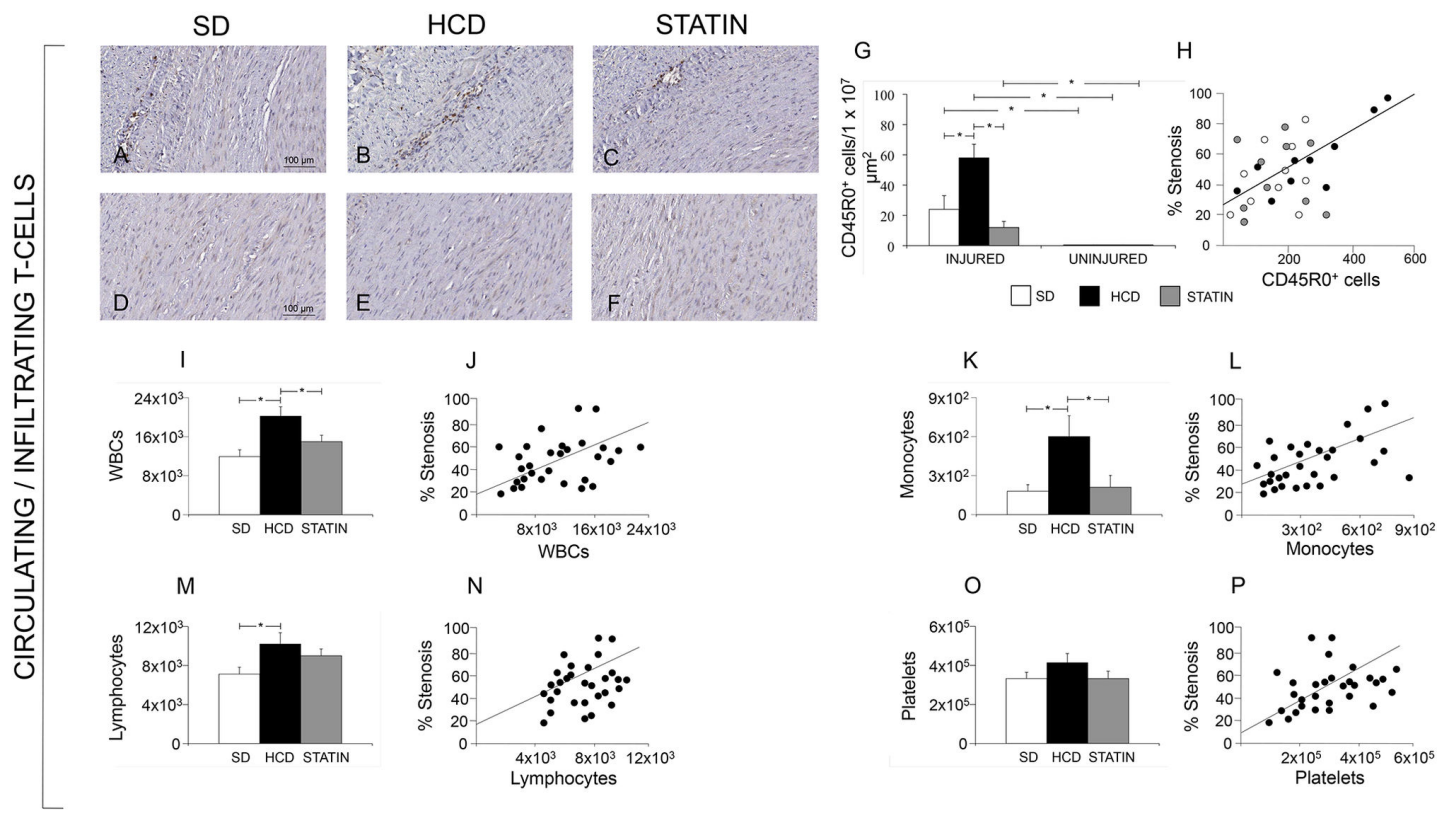
Atorvastatin significantly decreased circulating leukocytes and activated T-lymphocytes (CD45RO-positive) infiltrates in injured carotids. The number of circulating WBCs, monocytes and lymphocytes was increased by hypercholesterolemia compared to SD pigs (20215 ±1934/mm^3^ vs. 11924±1388/mm^3^, p=0.05, panel I; 600±160/mm^3^ vs. 180±50/mm^3^, p=0.04, panel K; and 10200±1160/mm^3^ vs. 7120±700/mm^3^, p=0.05, panel M, respectively). This significant difference was abolished by atorvastatin treatment. There was a correlation between circulating WBCs, monocytes, lymphocytes, platelets and the degree of stenosis (r=0.454 p=0.04, J; r=0.710 p=0.01, L; r=0.484 p=0.03, N; r=0.487 p=0.03, P). The CD45RO-positive cell infiltrates in injured carotids was significantly increased in HCD pigs compared to SD ones (58±9 vs. 249± cells/1x10^7^ μm^2^, p=0.05, panels A, B and G) while statin treatment reduced T-lymphocytes infiltration (12±4 vs. 58±9 cells/1x107 μm^2^, p=0.02, panels B, C and G). There were no CD45R0-positive cells in the contralateral uninjured carotid arteries (D-F). CD45R0-positive cells infiltration was positively correlated with the degree of stenosis (r=0.837, p=0.003, H).

Atorvastatin significantly reduced I/M Area and I/M Thickness ratio compared to HCD (Figure 6). The degree of stenosis was significantly greater in HCD compared to STATIN pigs. The deposition of ECM was significantly increased in HCD compared to SD pigs, both in the neointima and in the media (Figure 7). The deposition of ECM was significantly reduced in STATIN compared to HCD pigs, both in the neointima and in the media. Atorvastatin restored the expression of the protective genes HO-1 and iNOS at the site of vascular injury, enhancing iNOS expression also in contralateral uninjured arteries (Figure 8). HO-1 expression was reduced in the neointima and injured media of HCD compared to SD pigs. Atorvastatin treatment restored the expression of HO-1 to levels similar to SD group. There were no differences in contralateral uninjured arteries. There was a significant reduction of iNOS expression following vascular injury in the neointima of HCD compared to SD pig. Atorvastatin treatment significantly restored the expression of iNOS in neointima compared to HCD and induced a significant expression in the media of injured and contralateral arteries in comparison to HCD and SD. Atorvastatin significantly decreased circulating leukocytes and CD45RO-positive cell infiltrates in injured carotids (Figure 9). The number of circulating WBCs, monocytes and lymphocytes was increased by hypercholesterolemia compared to SD pigs. This significant difference was abolished by atorvastatin treatment. There was a correlation between circulating WBCs, monocytes, lymphocytes, platelets and the degree of stenosis. The activated T-lymphocytes cell infiltrates in injured carotids was significantly increased in HCD pigs compared to SD ones while statin treatment reduced T-lymphocytes infiltration. There were no activated T-lymphocytes in the contralateral uninjured carotid arteries. Activated T-lymphocytes infiltration was positively correlated with the degree of stenosis.

## Discussion

The present study demonstrates for the first time that statin treatment reduces generalized inflammation in spite of a minimal effect on lipids in a model of vascular injury in pigs fed high cholesterol diet. In fact diet is sufficient to cause a systemic inflammation by increasing the number of circulating WBCs, and to promote tissue-specific inflammation, by raising the amount of infiltrating leukocytes in WAT, liver and lung. These changes could contribute to increased vascular injury in hypercholesterolemic pigs. However statin treatment reversed not only enhanced vascular injury in hypercholesterolemic pigs but also attenuated most of systemic and localized inflammatory responses. 

Previous studies have indicated that the pig could represent an ideal preclinical model providing insight into lipoprotein metabolism for several reasons. In particular, pigs have omnivorous habits and a lipoprotein distribution similar to that found in humans; high-cholesterol diets are able to induce human-like changes in the plasma lipoprotein profile of pigs, with ~ 60% of plasma cholesterol distributed in LDL particles [25,26].

In the present study we set up a model of mild-hypercholesterolemic pig, with total and LDL cholesterol concentrations 20% higher than in normocholesterolemic pigs. These supra-physiological concentrations are similar to those of the vast majority of patients, very different from other models showing an increase of cholesterol up to 700%. In fact, nearly 50% of Western Countries adults have total cholesterol concentrations at the level that the National Cholesterol Education Program (NCEP) expert panel considers “borderline-high risk” [2].

Epidemiological studies have reported that leukocyte counts rise in atherosclerotic patients and there is a positive correlation between increased amounts of circulating leukocytes and coronary artery disease [27,28]. Transgenic and knockout mouse models in particular have proved useful to determine that the number of circulating leukocytes increase profoundly in atherosclerotic animals. Anyway, in these murine models the development of a leukocytosis similar to that seen in humans required the administration of a high-cholesterol diet with plasma cholesterol concentrations approaching 500-700 mg/dL [29–31]. Yet, the extent to which a slight increase of plasma cholesterol levels affects peripheral blood leukocytes remains to be thoroughly defined.

In this regard, blood analysis performed at sacrifice, after 16 weeks of hypercholesterolemic diet, displayed a significant increase in total WBCs, monocytes and lymphocytes in HCD-fed pigs compared to SD-fed pigs. This is in accordance with previous findings from our research group in which pigs fed a high-cholesterol diet showed an increased number of circulating WBCs, particularly monocytes [2]. The structural organization of WAT, liver and lung presents a highly vascularized milieu that allows a close interaction between metabolic and circulating immune cells. Chronic inflammatory reactions occurring in these tissues are characterized by a large infiltrate of lymphocytes that are mobilized to sites of injury where they produce cytokines that further activate macrophages and other inflammatory cells [1].

Previous studies in hypercholesterolemic mice have shown that macrophage and lymphocyte infiltration is of critical importance in WAT inflammation. T-lymphocytes may be enrolled during early adipose tissue inflammation preceding the appearance of macrophages, and the recruitment of these leukocytes to sites of inflammation is usually mediated by chemokines released from preadipocytes and adipocytes [32]. In addition, it has been shown that adipocyte hypertrophy coincides with the accumulation of T-cells and macrophages in mice fed a high fat diet [33,34]. Our results showed a significant increase of the mean cross-sectional area of adipocytes from HCD-fed pigs with a concomitant increase in the number of infiltrating T-lymphocytes, although in the absence of a significant increase in the amount of macrophages.

The occurrence of WAT inflammation in response to the excess of lipids leads to a low-level induction of inflammatory cytokines such as TNF-α, IL-1β and IL-6: this low-grade inflammation induces the further recruitment and activation of many professional immune cells [4]. In our model, we have observed a significant increase of circulating TNF-α and IFN-γ in mild-hypercholesterolemic pigs compared to normocholesterolemic pigs without significant alterations in IL-6 plasma concentration.

Metabolic disorders related to lipid metabolism such as the increase of serum triglycerides, total cholesterol and LDL-cholesterol, are well known causes of liver injury characterized by inflammation, steatosis and fibrosis [35]. HSCs play a pivotal role in this process. HSCs are vitamin A-storing cells, located in the perisinusoidal space of Disse. Chronic liver injury caused by an excessive intake of lipids can result in a pro-inflammatory hepatic environment with HSCs activation: HSCs start to proliferate in response to cytokines, stimulate the recruitment of inflammatory cells, and finally produce large amounts of ECM [36]. The immunohistochemical analysis of liver in our study displayed a 5-fold increase in the number of activated HSCs (α-SMA-positive cells) in HCD-fed pigs compared with SD-fed pigs. We have also found an increased expression of the stress-inducible genes HO-1 and iNOS in livers from mild-hypercholesterolemic pigs.

The administration of a high-cholesterol diet in our preclinical model did not induce either steatosis or fibrosis, HSCs activation notwithstanding. The evaluation of ECM deposition by trichrome staining and the immunohistochemical analysis specific for type I collagen, TGF-β1 and TGFβRII expression did not show significant differences on liver sections from HCD- and SD-fed pigs. Interestingly, HSCs activation and leukocyte infiltration of hepatic tissue did not alter hepatic function. In particular, HCD-fed pigs did not display increased levels of ALT and AST after 16 weeks on a high-cholesterol diet compared to SD-fed pigs. It is widely demonstrated that the administration of large amounts of dietary cholesterol causes hepatic inflammation in both mice and rabbits and it has been recently showed that high-fat feeding in mice increases the hepatic recruitment of T-lymphocytes and macrophages [5,37,38]. In addition, a previous study showed a correlation between plasma total cholesterol levels and the development of hepatic inflammation rather than steatosis [13]. Our work highlights that the leukocytes infiltrating the liver of HCD-fed pigs were primarily macrophages, but there was also an increased number of T- and B-lymphocytes. As already hypothesized in the case of WAT, T-lymphocytes may be responsible for the early changes that occur in the liver of hypercholesterolemic animals. The pro-inflammatory crosstalk between T-lymphocytes, hepatocytes and HSCs results in increased macrophage infiltration and consequently in chronic hepatic inflammation.

In our model, the inflammation extended beyond the WAT and the liver to the lung parenchyma, with a significantly increased amount of infiltrating macrophages in HCD-fed pigs compared to SD-fed pigs. This enrichment in lung macrophages was associated with neither parenchyma remodeling nor fibrosis. Moreover, no signs of increased type I collagen deposition as well as significant differences in the expression of TGF-β1 and TGFβRII were are observed. Our results are in contrast with a recent work from Naura et al. in which it has been shown that a high-fat diet induces the persistence of lung inflammation associated with tissue remodeling in apoE-KO mice [15].

To test whether reducing total cholesterolemia could determine a significant decrease of circulating leukocytes, a subset of HCD-fed pigs received high-dose atorvastatin (80mg/die) for 8 weeks. Statins, the inhibitors of HMG-CoA reductase, are extensively used in medical practice for their cholesterol-lowering effect. Large clinical trials have demonstrated that this class of drugs greatly reduces cardiovascular-related morbidity and mortality in patients with and without coronary disease. Several works have established that statins, in addition to their lipid-lowering effects, exert immuno-modulatory actions being able to decrease the expression of adhesion molecules, chemokines and chemokine receptors on both leukocytes and endothelial cells, ultimately limiting the recruitment of leukocytes across the vessel wall [39,40]. The conventional statin therapy dosage is ranging from 10 to 40mg but there are some studies reporting aggressive lipid-lowering therapy in patients with carotid artery disease demonstrating a reduced echo lucency of the plaques after high-dose stain treatment [41,42].

Consistent with above considerations, we determined that treatment of animals with high-dose atorvastatin resulted in a significant reduction of serum total cholesterol and LDL-cholesterol concentration. However, no significant reduction in triglycerides was noted.

Compared to the findings in HCD-fed pigs, treatment with atorvastatin markedly lowered the number of circulating WBCs, monocytes and lymphocytes. 

The immuno-modulatory effects of atorvastatin went beyond the reduction of circulating WBCs. Our findings demonstrated that high-dose atorvastatin treatment leads to a substantial decrease of the hypercholesterolemia-induced inflammatory infiltrates in WAT, liver and lung. In particular, treatment with atorvastatin in HCD-fed pigs prevented adipocyte hypertrophy limiting the number of infiltrating T-lymphocytes. Furthermore, statin-treatment considerably reduced the amount of circulating pro-inflammatory cytokines IFN-γ and TNF-α. In the liver, atorvastatin caused a significant reduction of activated HSCs and of the amount of infiltrating leukocytes, with a concomitant reduction of the stress-inducible gene HO-1 but without affecting the hepatic expression of iNOS. Last, atorvastatin treatment in HCD-fed pigs was effective in reducing the macrophage content in lung parenchyma.

Inflammatory response in liver, lung and adipose tissue correlated with enhanced intimal hyperplasia in carotid artery. Interestingly, no response was observed in contralateral uninjured artery. This highlights the importance of local injury and systemic/remote inflammation in the development of vascular pathology as we previously identified [2]. Atorvastatin treatment attenuated intimal hyperplasia and all its underlying mechanisms such as matrix deposition and activated T-lymphocytes infiltration. Restored expression of HO-1 and iNOS observed following atorvastatin treatment is another molecular event that indicate vascular benefit. Moreover, the atorvastatin-induced increased expression of iNOS in contralateral artery suggests that, besides systemic effects of statins, the local vascular effect could play an important role in vascular protection. 

In conclusion, our study demonstrates for the first time in a clinically relevant porcine model of diet induced hypercholesterolemia that even a moderate increase in plasma cholesterol levels can induce a significant increase in the amount of circulating WBCs. This effect extends beyond systemic inflammation to tissue-specific inflammation in WAT, liver and lung. We also establish that a mild-hypercholesterolemia is sufficient to induce an augmented leukocyte infiltrates in these organs. HCD-fed pigs treated with high-dose atorvastatin results in a reduction of total and LDL cholesterol and in markedly decreased systemic and tissue-specific inflammatory markers by preventing the development of an inflammatory milieu and the accumulation of infiltrating leukocytes.
